# Absolute and Body Mass Index Normalized Handgrip Strength Percentiles by Gender, Ethnicity, and Hand Dominance in Americans

**DOI:** 10.20900/agmr20200005

**Published:** 2019-12-31

**Authors:** Ryan McGrath, Kyle J. Hackney, Nicholas A. Ratamess, Brenda M. Vincent, Brian C. Clark, William J. Kraemer

**Affiliations:** 1 Department of Health, Nutrition, and Exercise Sciences, North Dakota State University, Fargo, ND 58108, USA; 2 Department of Health and Exercise Science, The College of New Jersey, Ewing, NJ 08628, USA; 3 Department of Statistics, North Dakota State University, Fargo, ND 58108, USA; 4 Ohio Musculoskeletal and Neurological Institute, Ohio University, Athens, OH 45701, USA; 5 Department of Biomedical Sciences, Ohio University, Athens, OH 45701, USA; 6 Department of Geriatric Medicine, Ohio University, Athens, OH 45701, USA; 7 Department of Human Sciences, The Ohio State University, Columbus, OH 43210, USA

**Keywords:** aging, epidemiology, hand strength, human development, muscle weakness

## Abstract

**Background:**

Gender and ethnicity are factors which influence strength, and hand dominance could be a critical component of handgrip strength (HGS) testing. Providing such HGS percentiles across the lifespan may help to identify weakness-related health concerns. We sought to generate growth charts and curves for HGS by gender and ethnicity in a nationally-representative sample of Americans aged 6–80 years.

**Methods:**

Data from 13,617 participants in the 2011–2012 and 2013–2014 waves of the National Health and Nutrition Examination Survey were analyzed. HGS was measured with a handgrip dynamometer. Age, gender, ethnicity, and hand dominance were self-reported. Body Mass Index (BMI) was calculated from height and body mass. Measures of absolute HGS and HGS normalized to BMI were separately included in parametric quantile regression analyses for determining the 10th–90th percentiles across ages by gender and ethnicity. Similar models were also conducted by hand dominance.

**Results:**

Differences in absolute HGS and HGS normalized to BMI quantiles across ages existed for each ethnicity regardless of gender. In men, absolute HGS generally increased until about 25 years of age, began to decline around age 30 years, and regressed into older adulthood. In women, absolute HGS appeared to rise starting at age 6 years, peaked between 20 and 30 years of age, but was maintained into mid-life before declining in older adulthood. Similar results were found for HGS normalized to BMI.

**Conclusions:**

Our findings provide percentile charts for HGS capacity that could be utilized for comparing individual measures of HGS to those from a United States population-representative sample.

## INTRODUCTION

Muscle strength can be conveniently assessed by measuring maximal handgrip strength (HGS) with a handgrip dynamometer [1]. Those that have lower levels of muscle strength earlier in life often experience poor health outcomes related to weakness later in life [2]. Similarly, health consequences related to low muscle strength, as measured by HGS, occur throughout the lifespan [3]. For example, the presence of pediatric sarcopenia, as characterized by low skeletal muscle mass and function at childhood, places children at higher risk for several adverse health outcomes [4]. Low muscle strength in adolescents is linked to psychiatric diagnoses, suicide, cardiovascular diseases, and all-cause mortality at young adulthood [5]. Leong et al. [6] also found that every 5-kilogram decrease in HGS was associated with 16% greater risk for all-cause mortality at a median 4-year follow-up in 142,861 adults aged 35–70 years. The 2018 Physical Activity Guidelines for Americans highlight the importance of participating in muscle-strengthening activities for health at childhood, adolescents, adulthood, and older adulthood [7]. Moreover, strength capacity is a subdomain in the intrinsic capacity construct [8], and handgrip strength is part of decision algorithms for diagnosing sarcopenia [9]. Therefore, muscle strength should be considered a critical health indicator across the lifespan.

Gender and ethnicity are important factors that may influence muscle strength. For example, Silva et al. [10] showed that lifespan skeletal muscle mass was higher in men than women, and non-Hispanic Blacks had the highest skeletal muscle mass, followed by non-Hispanic Whites, Hispanics, and non-Hispanic Asians. These muscle mass differences may help to explain why HGS thresholds for determining weakness have emphasized stratification by gender and ethnicity [11–13]. Additional research is also recommended for developing predictive regression models for HGS that emphasize age, gender, and ethnicity [14]. Similarly, as HGS protocols continue evolving, examining the HGS of the dominant and non-dominant hands may provide important insights into how HGS is measured and data are used, especially because handedness changes throughout the lifespan [2,15]. Given the positive health aspects of muscle strength over the lifespan, along with the role gender and ethnicity have on strength capacity, assessments of strength could be individualized to gender and ethnicity. Creating lifespan muscle strength centiles by gender and ethnicity across ages will help to provide a detailed norm-referenced standard for comparing individual HGS measures to those from an American population-representative sample. Further, normative data for HGS is a high research priority for developing validated cut-points that improve how we diagnose sarcopenia [9]. The purpose of this study was to generate growth charts and curves for HGS by gender and ethnicity in a nationally-representative sample of Americans aged 6–80 years.

## MATERIALS AND METHODS

### Participants

Secondary analyses of data from participants in the 2011–2012 and 2013–2014 waves of the National Health and Nutrition Examination Survey (NHANES) were performed for this investigation. The NHANES is designed to assess the health and nutrition status of American children, adolescents, and adults [16]. Trained interviewers performed health interviews in respondents’ homes using computer-aided interview systems and participants visited mobile examination centers for enhanced assessments. Interviewers and mobile examination centers traveled to locations throughout the United States to diversify sampling [17].

Oversampling for those aged at least 60-years, Hispanics, non-Hispanic Asians, non-Hispanic Blacks occurred to create reliable data that represented all ages and ethnicities in the United States [16]. Overall interview response rates for the NHANES were relatively high for each waved included in our analyses [18]. The NHANES uses an intricate, 4 stage probability sampling design to produce a representative sample of non-institutionalized persons in the United States. Written informed consent was provided by participants and NHANES protocols were approved by the National Center for Health Statistics Research Ethics Review Board (Protocol #2011-17).

### Measures

A digital handgrip dynamometer (Takei Dynamometer Model T.K.K.5401; Akiha-Ku, Japan) was used to measure HGS. All personnel that administered HGS tests received training and calibration procedures, and a calibration log. Before testing, participants were asked about their ability to complete HGS protocols, including if they had surgery to the hand or wrist within the previous 3 months that prohibited them from HGS testing, or if they were unable to hold the dynamometer with either hand. Interviewers verbally explained and demonstrated the HGS protocols. Participants were instructed to stand with their feet hip width apart and hold the dynamometer away from their body and in line with the forearm, at thigh level so that the dynamometer did not touch the body (unless physically unable). An emphasis was made on squeezing the handle of the dynamometer hard and quickly [19].

A practice trial at sub-maximal effort was performed by participants to determine if the dynamometer was properly fitted to their hand size and to confirm understanding of the HGS protocol. Each person squeezed the dynamometer with maximal effort, exhaling while squeezing, and then released the muscle contractions. Participants reported their hand dominance and the decision to begin HGS testing on the dominant or non-dominant hand was randomized. Each hand was tested 3 times, alternating hands between trials, with 60 seconds of rest between measures on the same hand. The highest recorded HGS value on either hand was included in the analyses. More details about the HGS protocols are published elsewhere [20,21].

**Figure 1.**
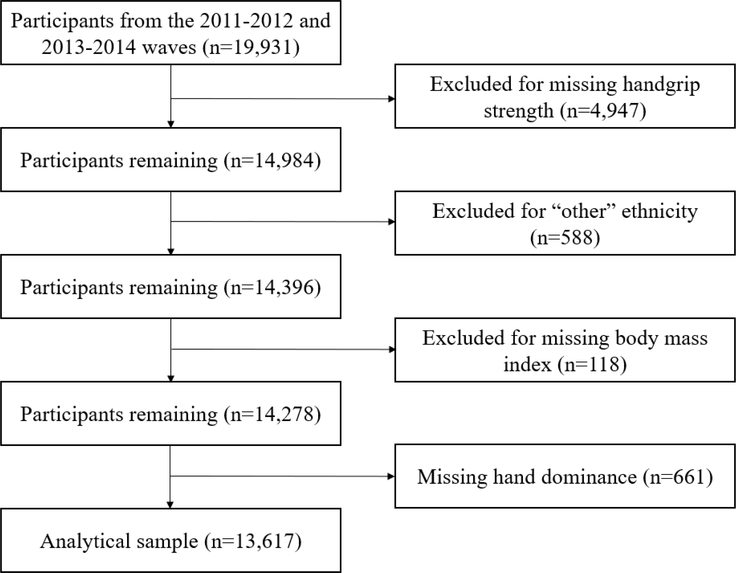
Diagram of Participant Exclusions.

Age, gender, and ethnicity (Hispanic, non-Hispanic Asian, non-Hispanic Black, non-Hispanic White) were self-reported. A fixed stadiometer was used to collect standing height and a digital scale (Mettler-Toledo International; Columbus, OH) was used to collect body mass. Body mass index (BMI) was calculated as body mass in kilograms divided by height in meters-squared. Given that body composition may impact strength capacity, we included both absolute HGS and HGS normalized to BMI (HGS normalized to BMI = HGS (kg)/BMI (kg/m^2^)) in our analyses [14]. A diagram for participant exclusions is presented in Figure 1.

### Statistical Analysis

All analyses were conducted with SAS 9.4 software (SAS Institute; Cary, NC, USA). Parametric quantile regression models were run to generate absolute HGS and HGS normalized to BMI growth charts for the 10th–90th percentiles by gender and ethnicity for Americans aged 6–80 years. The same procedures were also performed for HGS data from the dominant and non-dominant hands. These 10 quantiles were pre-specified by investigators to bolster the interpretability of the results in the free-living environment. Fitted values for absolute HGS and HGS normalized to BMI were estimated at each ascertained age. Utilizing parametric quantile regression to create growth curves allows the models to fit conditional quantiles of strength capacity without assuming a parametric distribution, estimate the entire conditional distribution of the response, and reveal the effects of predictors on various parts of the response distribution, thereby making quantile regression feasible for analyses of datasets with larger amounts of observations such as NHANES [22].

The PROC QUANTREG procedure was used to determine the gender- and ethnicity-specific growth patterns of absolute HGS and HGS relative to BMI with 6 powers of age (age, age^2^, age^3^, age^−1^, √age, √age × age) [23]. Sample weights were utilized in the PROC QUANTREG procedure to acknowledge the NHANES sampling methods and generate an unbiased national estimate [24]. The PROC SGPLOT procedure created the absolute HGS and HGS relative to BMI growth curves by gender and ethnicity across ages from each quantile regression output [25].

## RESULTS

**Figure 2.**
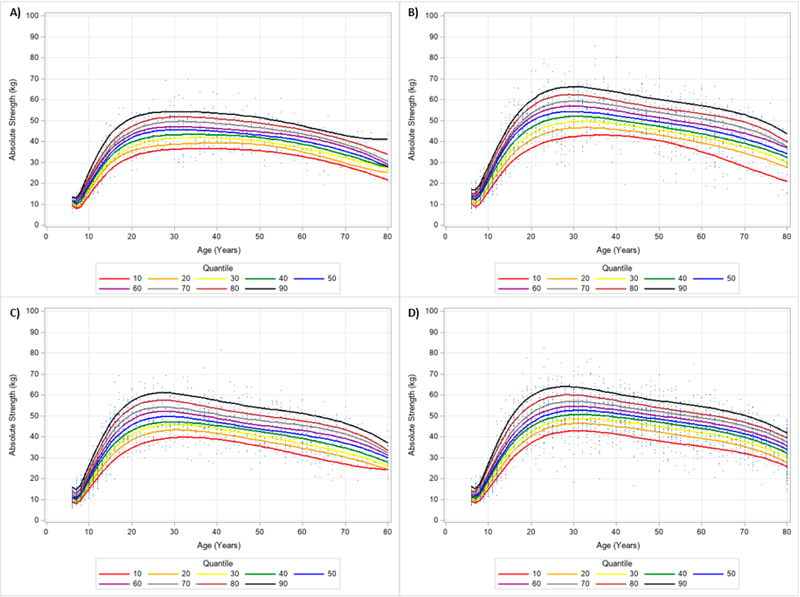
Absolute Handgrip Strength Centile Curves with Polynomials and Scatterplots for Men by Ethnicity. (**A**) non-Hispanic Asian; (**B**) non-Hispanic Black; (**C**) Hispanic; (**D**) non-Hispanic White. Note: kg = kilograms.

**Figure 3.**
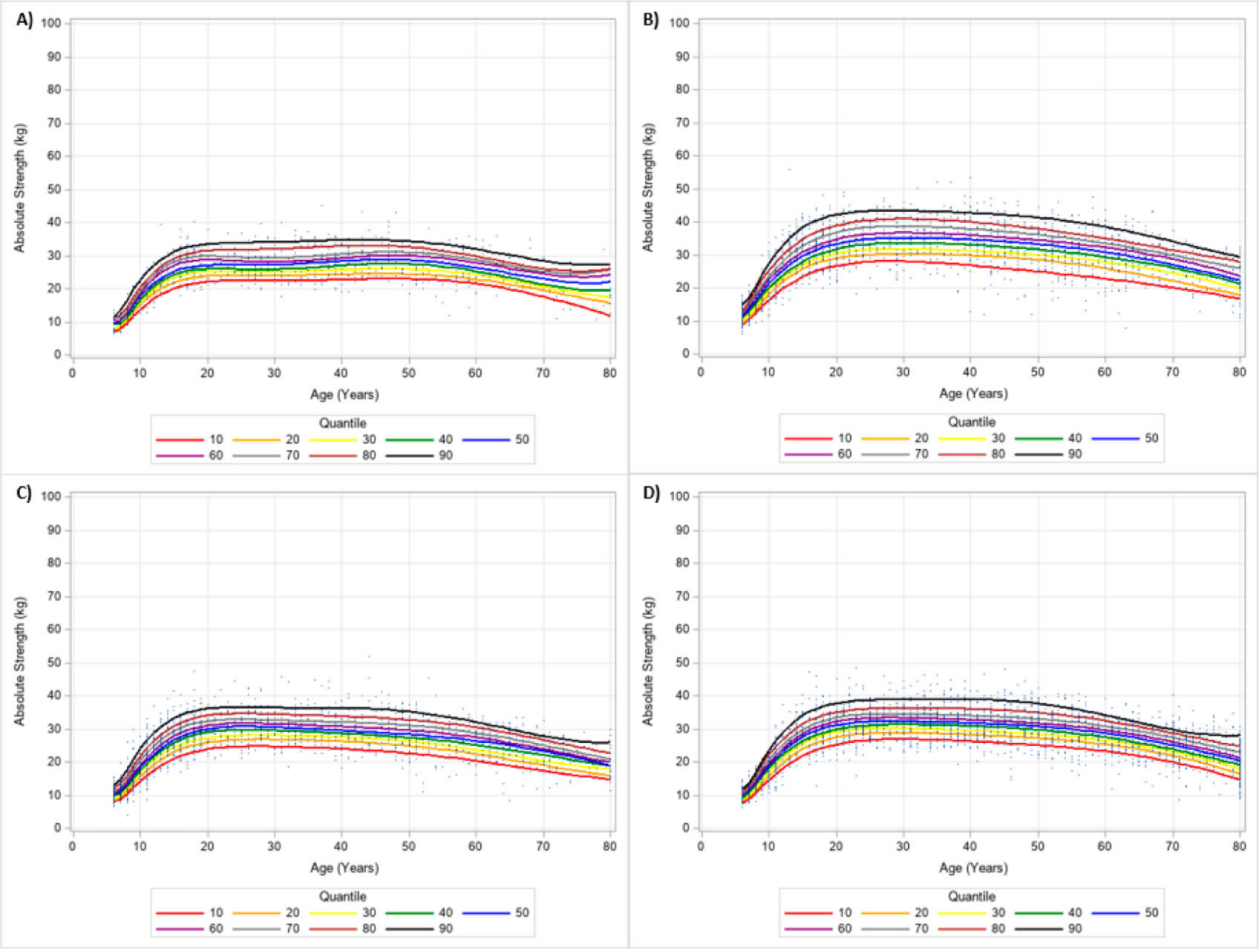
Absolute Handgrip Strength Centile Curves with Polynomials and Scatterplots for Women by Ethnicity. (**A**) non-Hispanic Asian; (**B**) non-Hispanic Black; (**C**) Hispanic; (**D**) non-Hispanic White. Note: kg = kilograms.

The descriptive characteristics of the 13,617 participants (*n* = 6723 (49.4%) men (age range: 6–80 years); *n* = 6894 (50.6%) women (age range: 6–80 years)) included are in Table 1. To make comparisons for the descriptive characteristics by gender and ethnicity, the means and 95% confidence intervals are in Supplementary File 1. Table 2 shows the number of participants by age group for each gender and ethnicity. Figures 2 and 3 show the 10 percentile curves for absolute HGS with polynomials together and scatterplots by ethnicity for men and women, respectively. Differences in absolute HGS quantiles across ages existed for each ethnicity regardless of gender. In men, there were mostly rapid increases in absolute HGS beginning at 7 years of age until about 25 years of age. Declines in absolute HGS appeared to occur at around 30 years of age and accelerated reductions in absolute HGS seemingly started at about 70 years of age. Absolute HGS looked to rise starting at 6 years of age and peaked between 20–30 years of age for women. Non-Hispanic Asian women appeared to uniquely maintain their peak absolute HGS into mid-life (i.e., 40 years of age). Table 3 shows an abbreviated amount of information for absolute HGS quantiles by age group, gender, and ethnicity, while Supplementary File 2 presents specific absolute HGS quantile values for each age, gender, and ethnicity.

**Figure 4.**
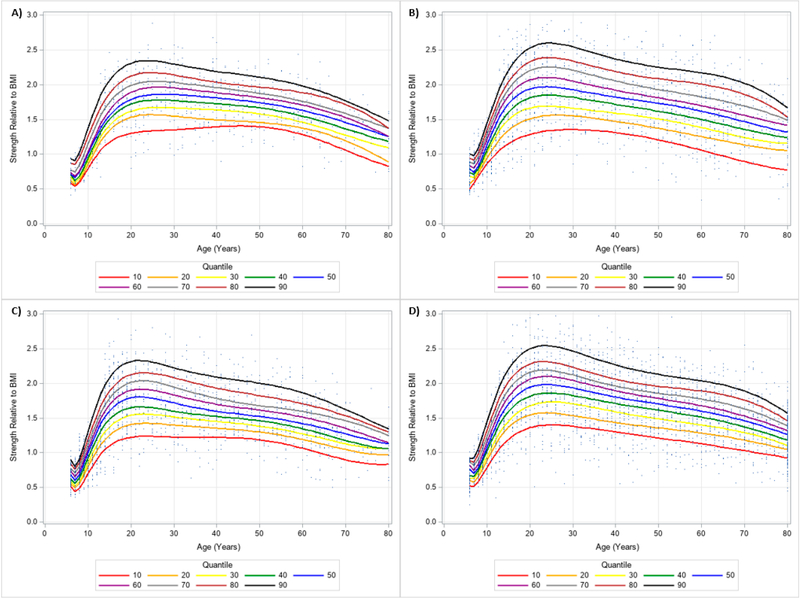
Handgrip Strength Normalized to Body Mass Index Centile Curves with Polynomials and Scatterplots for Men by Ethnicity. (**A**) non-Hispanic Asian; (**B**) non-Hispanic Black; (**C**) Hispanic; (**D**) non-Hispanic White. Note: kg = kilograms.

**Figure 5.**
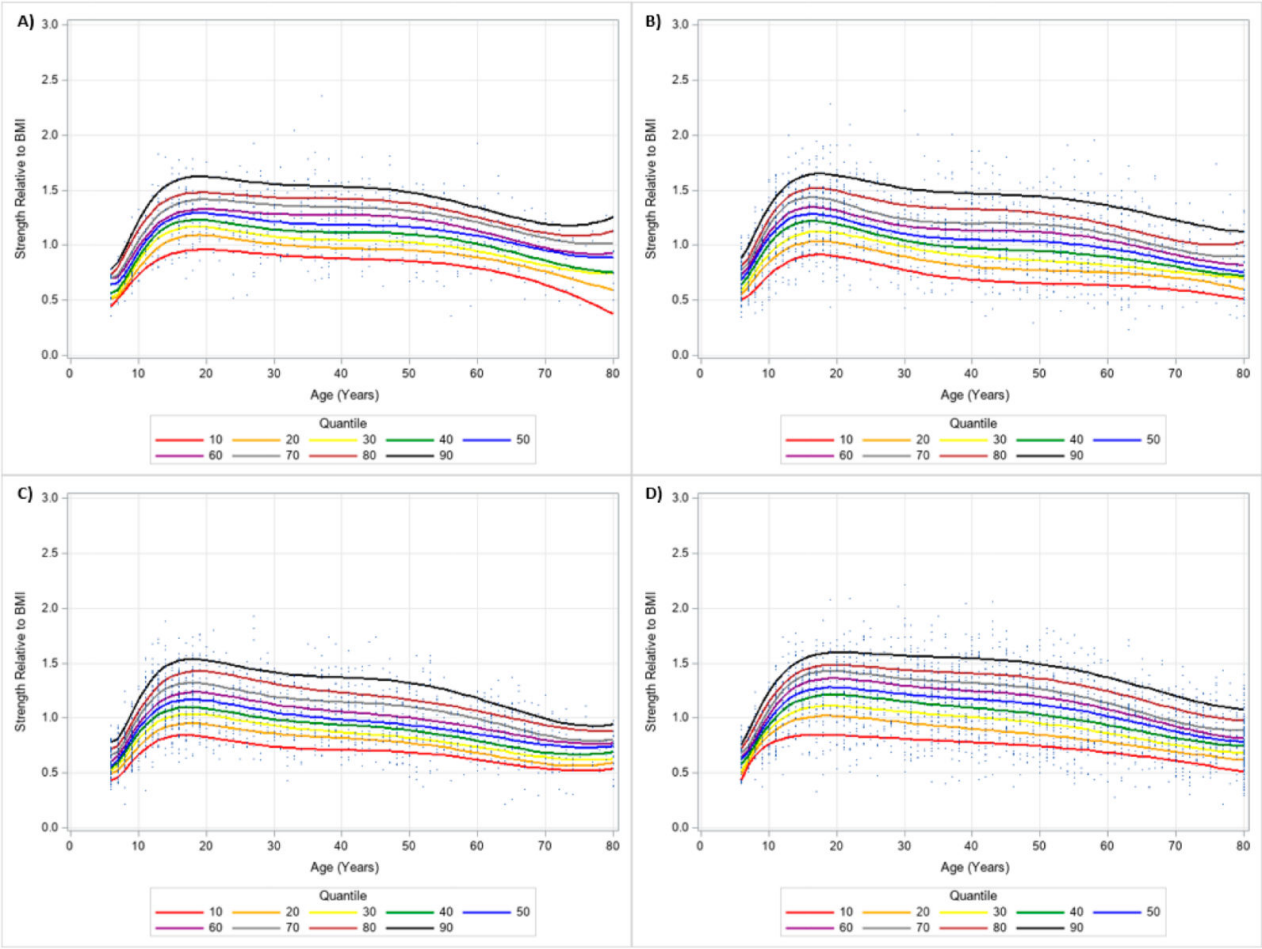
Handgrip Strength Normalized to Body Mass Index Centile Curves with Polynomials and Scatterplots for Women by Ethnicity. (**A**) non-Hispanic Asian; (**B**) non-Hispanic Black; (**C**) Hispanic; (**D**) non-Hispanic White. Note: kg = kilograms.

The 10 percentile curves for HGS relative to BMI with polynomials together and scatterplots by ethnicity for men are women are presented in Figures 4 and 5, respectively. There were differences in HGS normalized to BMI quantiles across ages for each ethnicity irrespective of gender. After a small dip in BMI normalized HGS from age 6–7 years in men, HGS normalized to BMI appeared to increase at 7 years and peaked at about 23 years of age, but began to decline at around 25 years of age for each ethnicity. Interestingly, HGS relative to BMI generally increased starting at 6 years of age but peaked before 20 years of age for women. This was then followed by observed reductions of BMI adjusted HGS occurring after 20 years of age. Table 4 presents an abbreviated amount of information for BMI normalized HGS quantiles for each age, gender, and ethnicity; whereas, Supplementary File 3 provides specific HGS normalized to BMI quantile values for each age, gender, and ethnicity.

**Figure 6.**
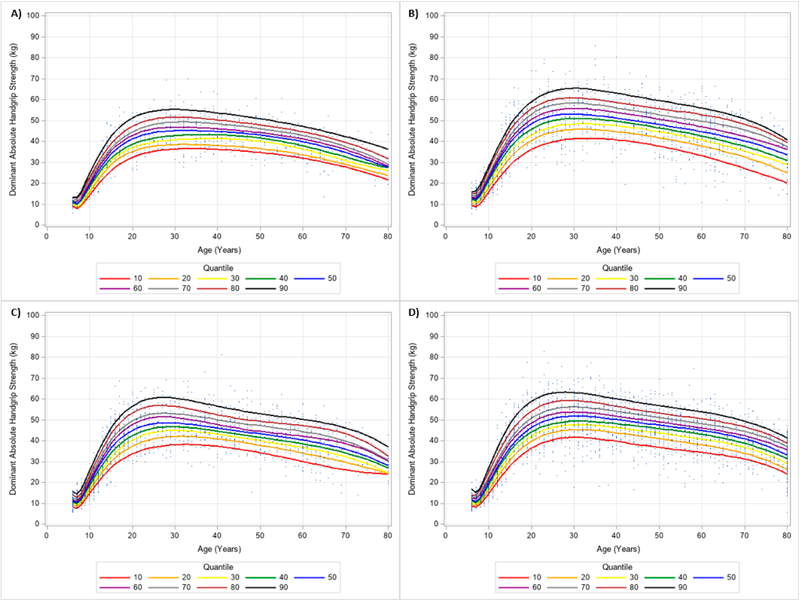
Absolute Dominant Handgrip Strength Centile Curves with Polynomials and Scatterplots for Men by Ethnicity. (**A**) non-Hispanic Asian; (**B**) non-Hispanic Black; (**C**) Hispanic; (**D**) non-Hispanic White. Note: kg = kilograms.

**Figure 7.**
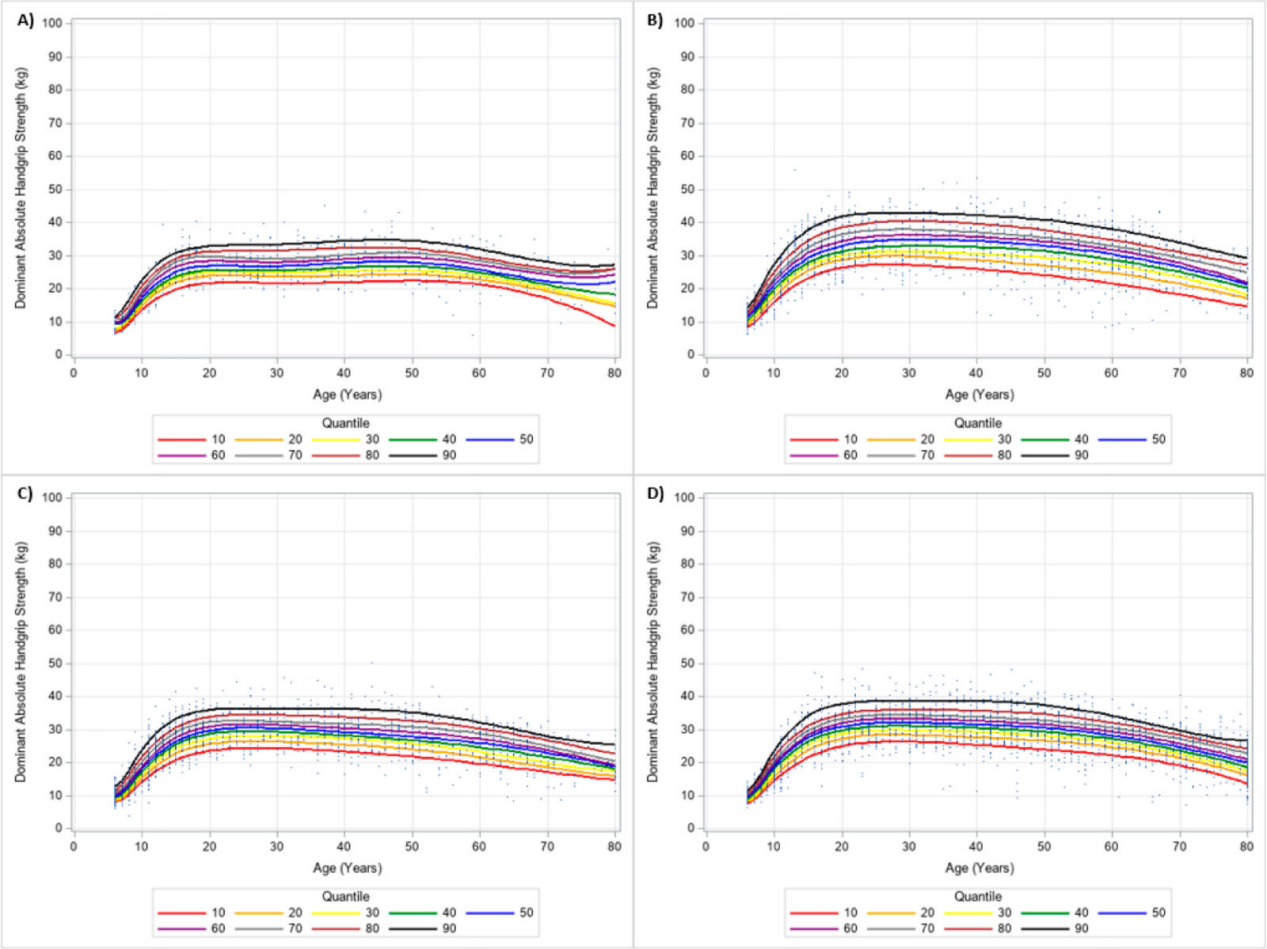
Absolute Dominant Handgrip Strength Centile Curves with Polynomials and Scatterplots for Women by Ethnicity. (**A**) non-Hispanic Asian; (**B**) non-Hispanic Black; (**C**) Hispanic; (**D**) non-Hispanic White. Note: kg = kilograms.

**Figure 8.**
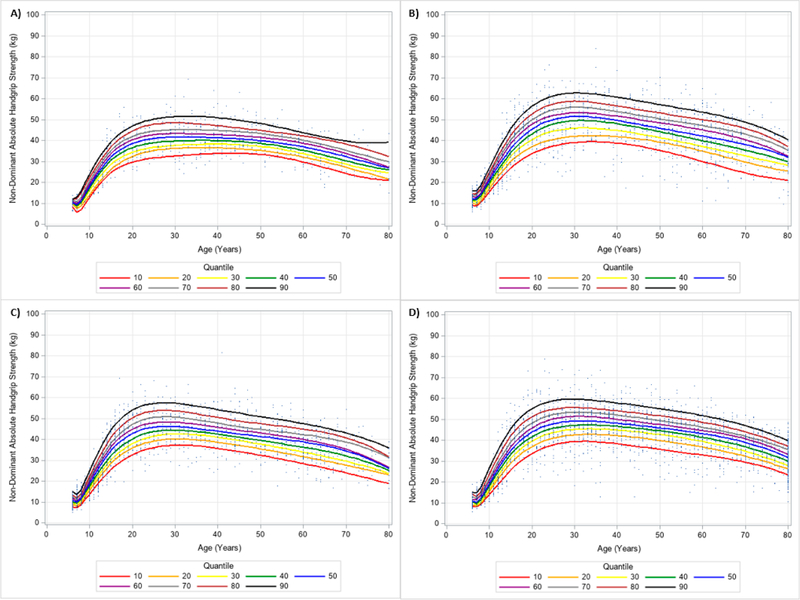
Absolute Non-Dominant Handgrip Strength Centile Curves with Polynomials and Scatterplots for Men by Ethnicity. (**A**) non-Hispanic Asian; (**B**) non-Hispanic Black; (**C**) Hispanic; (**D**) non-Hispanic White. Note: kg = kilograms.

**Figure 9.**
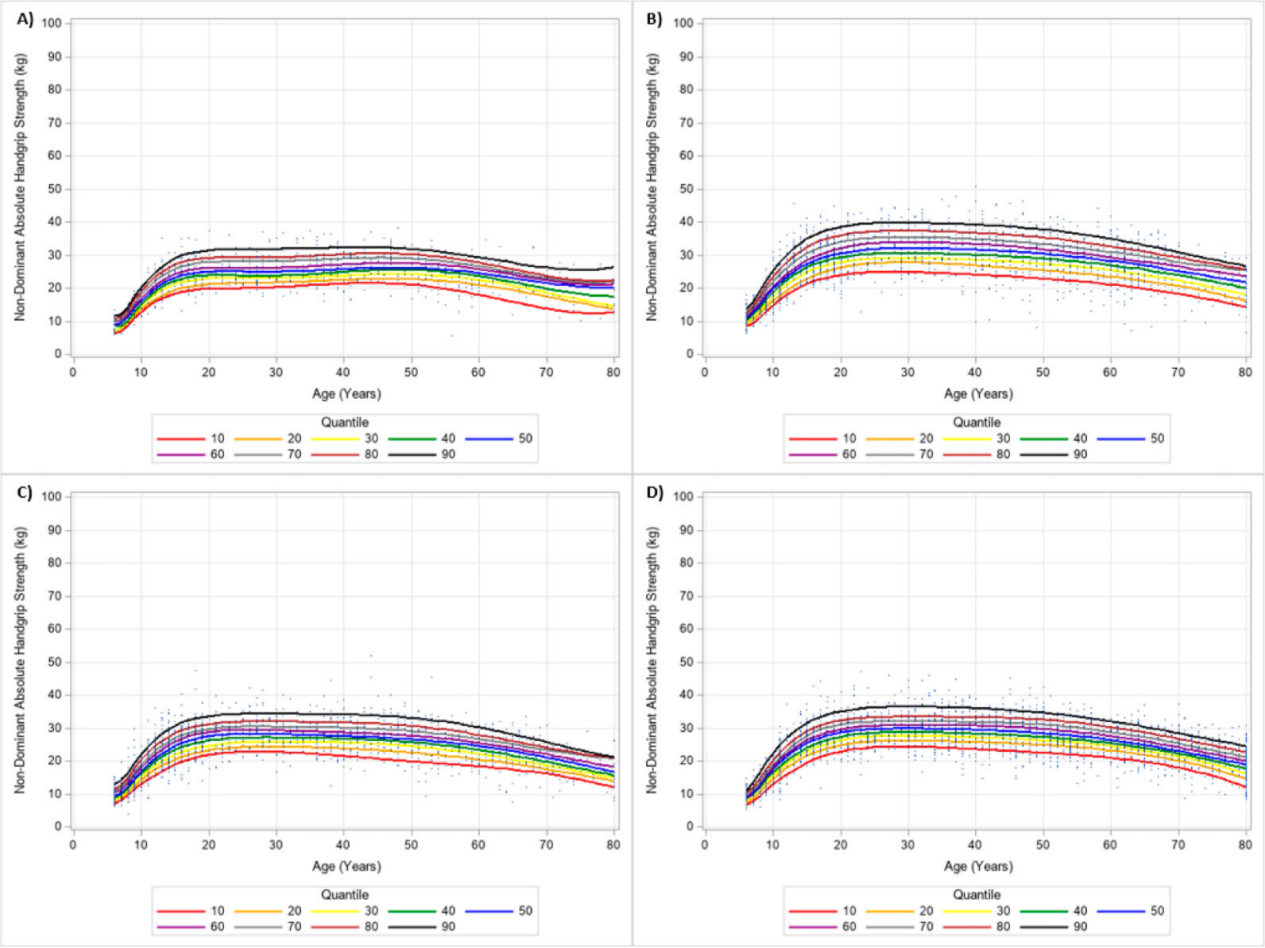
Absolute Non-Dominant Handgrip Strength Centile Curves with Polynomials and Scatterplots for Women by Ethnicity. (**A**) non-Hispanic Asian; (**B**) non-Hispanic Black; (**C**) Hispanic; (**D**) non-Hispanic White. Note: kg = kilograms.

**Figure 10.**
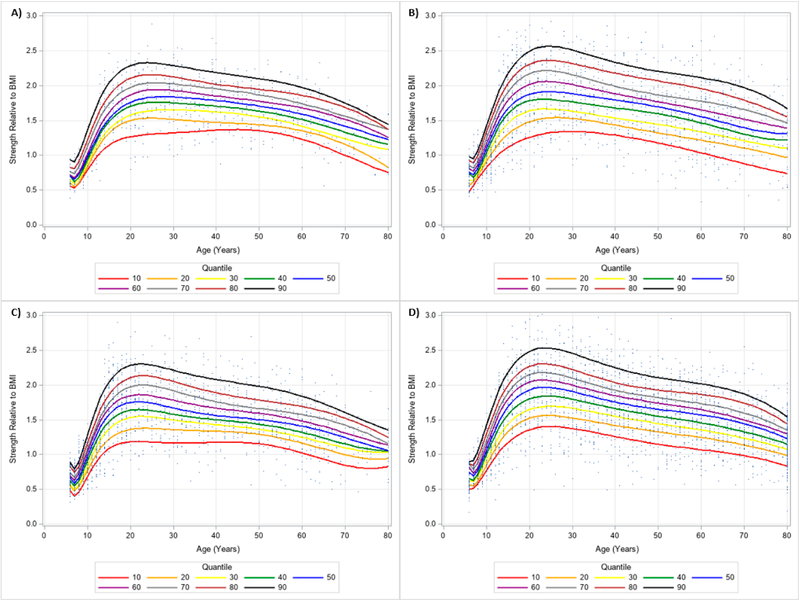
Dominant Handgrip Strength Normalized to Body Mass Index Centile Curves with Polynomials and Scatterplots for Men by Ethnicity. (**A**) non-Hispanic Asian; (**B**) non-Hispanic Black; (**C**) Hispanic; (**D**) non-Hispanic White. Note: kg = kilograms.

**Figure 11.**
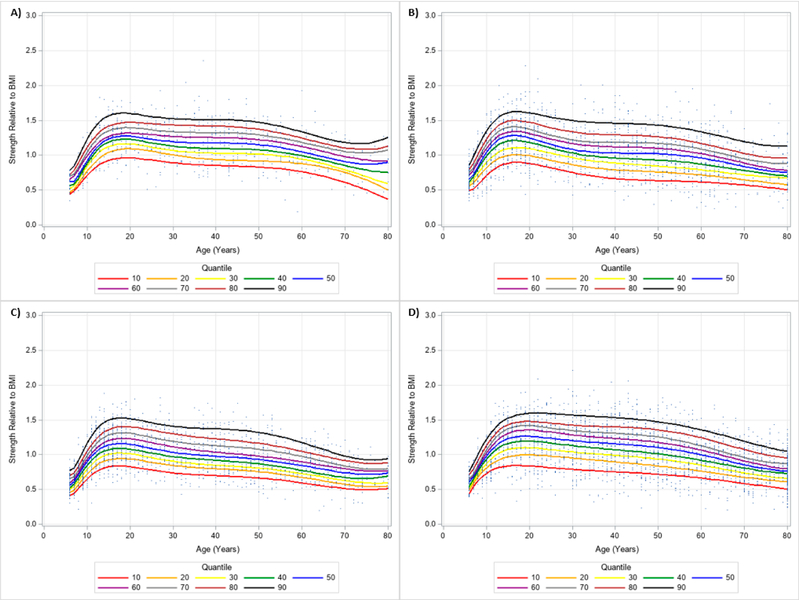
Dominant Handgrip Strength Normalized to Body Mass Index Centile Curves with Polynomials and Scatterplots for Women by Ethnicity. (**A**) non-Hispanic Asian; (**B**) non-Hispanic Black; (**C**) Hispanic; (**D**) non-Hispanic White. Note: kg = kilograms.

**Figure 12.**
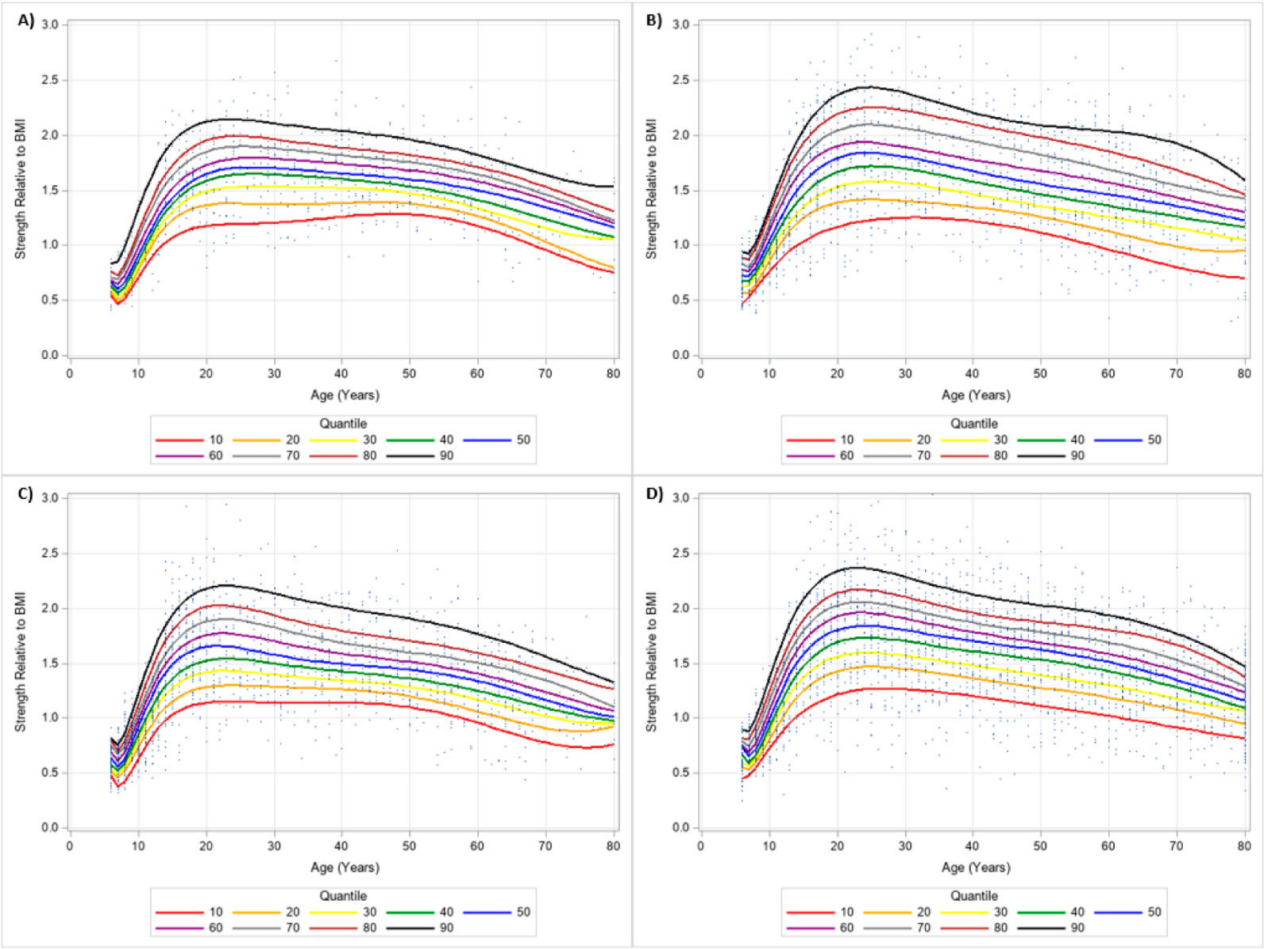
Non-Dominant Handgrip Strength Normalized to Body Mass Index Centile Curves with Polynomials and Scatterplots for Men by Ethnicity. (**A**) non-Hispanic Asian; (**B**) non-Hispanic Black; (**C**) Hispanic; (**D**) non-Hispanic White. Note: kg = kilograms.

**Figure 13.**
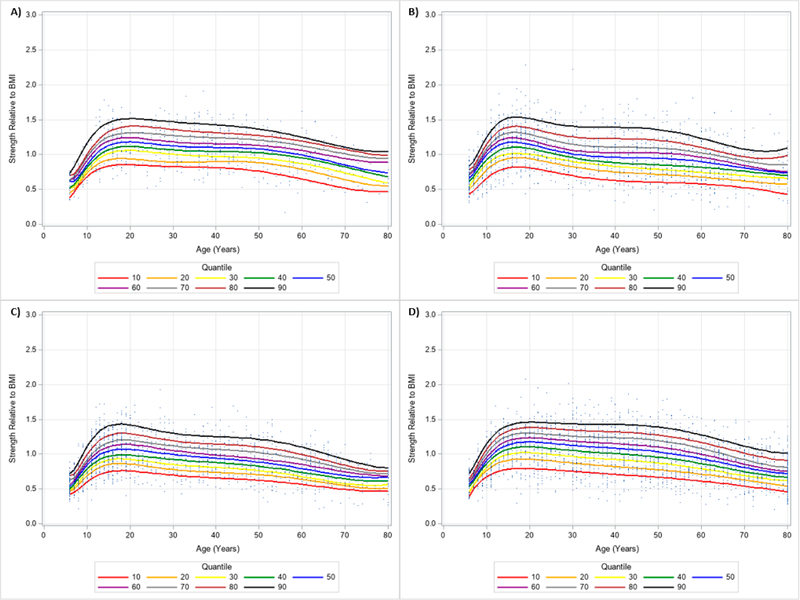
Non-Dominant Handgrip Strength Normalized to Body Mass Index Centile Curves with Polynomials and Scatterplots for Women by Ethnicity. (**A**) non-Hispanic Asian; (**B**) non-Hispanic Black; (**C**) Hispanic; (**D**) non-Hispanic White. Note: kg = kilograms.

Similar results were observed when evaluating the dominant and non-dominant absolute HGS and BMI normalized HGS quantiles. HGS was generally stronger on the dominant hand compared to the dominant hand, regardless of gender and ethnicity. Figures 6 and 7 present the 10 percentile curves for absolute dominant HGS with polynomials together and scatter plots by ethnicity for men and women, respectively; whereas, Figures 8 and 9 show the 10 percentile curves for absolute non-dominant HGS with polynomials together and scatter plots by ethnicity for men and women, respectively. Table 5 shows an abbreviated amount of information for dominant absolute HGS quantiles by gender and ethnicity (full results are in Supplementary File 4); whereas, Table 6 presents an abbreviated amount of information for non-dominant absolute HGS quantiles by gender and ethnicity (full results are in Supplementary File 5). The 10 percentile curves for dominant HGS normalized to BMI with polynomials together and scatter plots by ethnicity for males and females are in Figures 10 and 11, respectively, while the 10 percentile curves for non-dominant HGS normalized to BMI with polynomials together and scatter plots by ethnicity for males and females are in Figures 12 and 13, respectively. Table 7 displays an abbreviated amount of information for BMI normalized dominant HGS quantiles by gender and ethnicity (full results are in Supplementary File 6) and Table 8 shows an abbreviated amount of information for BMI normalized non-dominant HGS quantiles by gender and ethnicity (full results are in Supplementary File 7).

## DISCUSSION

This investigation provided absolute HGS and HGS normalized to BMI percentiles by gender and ethnicity for Americans aged 6–80 years. Individuals could use these HGS centiles for comparing their HGS capacity to their American peers. Acknowledgement should be given to how gender and ethnicity factor into strength capacity throughout the lifespan, and how health outcomes from low muscle strength impact each gender and ethnicity differently. Moreover, utilizing both absolute HGS and HGS normalized to BMI in assessments of strength capacity will provide more detailed insights into how body composition influences muscle strength.

While our investigation provided HGS centiles for Americans aged 6–80 years by gender and ethnicity, other investigations have found similar results in cohorts from other countries. Kenny et al. [26] provided percentiles for absolute HGS by gender for Irish adults aged 50–85 years, whereas Dodds et al. [27] revealed normative centiles for British persons aged 4–90 years. HGS values for Canadians aged 6–79 years are also comparable to our findings [28]. Further, our findings parallel with those from Peterson and Krishnan [29], who provided gender-specific HGS percentiles for Americans aged 6–80 years with NHANES data. Although our results mostly align with previous investigations that have evaluated gender-specific HGS centiles at different ages [19,26,27,29,30], our findings show that ethnicity is also a factor that influences strength in Americans during the lifespan. The differences we observed in our HGS percentiles for each ethnicity support those who suggest demographic characteristics such as ethnicity need to be considered for risk stratification related to weakness and interventions aiming to improve strength capacity [31]. Therefore, we agree with previous conclusions that suggest different standards for skeletal muscle and strength capacity should be applicable to each ethnicity [10,14].

Although absolute HGS provides easily interpretable information that is associated with a variety of health outcomes [2], body composition also factors into muscle strength and should be accounted for when evaluating HGS [14,32]. Our findings also provide HGS normalized to BMI quantiles by gender and ethnicity for Americans aged 6–80 years. While muscle strength changes throughout the life course for each gender and ethnicity, BMI also changes [33]. For example, BMI is a surrogate measure of body fat that is often used in population-based studies [34], and the higher BMI seen at older age is often attributed to age-related reductions in muscle mass and increases in adipose tissue [35]. The results of our investigation showed that measures of absolute HGS mostly tended to peak at around 30 years of age regardless of gender and ethnicity, but measures of HGS relative to BMI peaked earlier in life, especially for women (i.e., before 20 years of age). This further demonstrates the importance of acknowledging the role of body composition on strength capacity. Our findings are also in agreement with those from Wang et al. [36] such that HGS is generally stronger in the dominant hand compared to the non-dominant hand; however, handedness may change over the lifespan [15], and HGS can also be greater in the non-dominant relative to the dominant hand at older age [36]. We recommend that both absolute HGS and HGS normalized to BMI be evaluated for any thresholds and centiles of HGS, and consideration be given to what hand is being measured during HGS testing. We also recommend that our results be used to help develop validated cut-off points for appropriate sarcopenia diagnoses [9].

Some limitations of the reported data should be acknowledged. Although our HGS centiles are generalizable for Americans, the HGS protocols in the NHANES may differ from other HGS protocols, which in turn, may influence HGS values. The dip seen from 6–7 years of age for our quantiles may be best explained by model fitting in our analyses. Sampling was also higher for certain ages, genders, and ethnicities than others. Although HGS was used in this investigation for measuring muscle strength, data for other measures of strength capacity such as knee extension strength were not available in the NHANES and could not be analyzed. While HGS normalized to BMI helps to account for the role of body composition in strength capacity, it is challenging to determine if differences in HGS relative to BMI across the lifespan are due to changes absolute HGS, BMI, or both. Nevertheless, the use of BMI is still recommended when normalizing HGS to a measure of body composition [14]. We recommend that HGS centiles continue to be monitored and updated in Americans of all ages, genders, and ethnicities, including the creation of HGS centiles in special (e.g., persons with disabilities) and morbid populations. Evaluating HGS centiles for morbid populations may help to distinguish strength capacity in populations that are “healthy” and “not-healthy”.

## CONCLUSIONS

Our findings provide percentile charts for absolute HGS and HGS normalized to BMI by gender and ethnicity for Americans aged 6–80 years. The role of age, gender, and ethnicity should be acknowledged when evaluating strength capacity. Consideration should also be given to the hand being measured during HGS testing. We recommend that health practitioners working with persons of all ages utilize measures of HGS for determining strength capacity, and compare their HGS values to the appropriate population-representative growth charts. Understanding such information may help to better identify those at risk for future weakness. Changing strength capacity trajectories through interventions aiming to improve muscle strength may help to prevent adverse health outcomes associated with low muscle strength.

## Supplementary Material

1

2

3

4

5

6

7

## Figures and Tables

**Table 1.** Descriptive Characteristics of the Participants.

Variables	Age (years)	Absolute HGS (kg)	BMI (kg/m^2^)	Right Handed (*n* (%))
*Men*				

Non-Hispanic Asian (*n* = 827)	35.1 ± 20.2	37.3 ± 11.9	23.5 ± 4.7	760 (91.9%)
Non-Hispanic Black (*n* = 1718)	34.4 ± 22.5	40.6 ± 15.1	26.1 ± 7.6	1494 (86.9%)
Hispanic (*n* = 1666)	30.5 ± 21.1	35.8 ± 14.4	25.7 ± 6.6	1544 (92.6%)
Non-Hispanic White (*n* = 2512)	40.8 ± 23.2	40.9 ± 13.8	26.7 ± 7.0	2282 (90.8%)

*Women*				

Non-Hispanic Asian (*n* = 826)	35.0 ± 20.0	25.1 ± 6.5	22.7 ± 4.9	777 (94.0%)
Non-Hispanic Black (*n* = 1800)	34.6 ± 22.1	28.7 ± 8.5	28.6 ± 9.2	1629 (90.5%)
Hispanic (*n* = 1737)	31.6 ± 21.4	24.9 ± 7.4	26.6 ± 7.5	1633 (94.0%)
Non-Hispanic White (*n* = 2531)	42.6 ± 23.0	27.1 ± 7.2	27.4 ± 8.0	2320 (91.6%)

Note: Results are presented as mean ± standard deviation or frequency (percentage) where indicated. BMI = body mass index; HGS = handgrip strength; kg = kilograms; kg/m^2^ = kilograms per meters-squared.

**Table 2.** Frequencies of Participants by Age Group for Each Gender and Ethnicity.

Variables	6–9 Years	10–19 Years	20–29 Years	30–39 Years	40–49 Years	50–59 Years	60–69 Years	70–79 Years	80+ Years
*Men*									

Non-Hispanic Asian	82	167	120	118	118	99	78	33	12
Non-Hispanic Black	223	433	209	161	160	187	223	91	31
Hispanic	266	491	184	160	166	152	176	52	19
Non-Hispanic White	232	408	301	338	299	294	239	217	184

*Women*									

Non-Hispanic Asian	79	176	110	128	118	96	73	36	10
Non-Hispanic Black	226	438	214	159	204	220	213	91	35
Hispanic	247	510	186	182	182	152	183	73	22
Non-Hispanic White	174	378	287	342	317	309	280	257	187

**Table 3.** Abbreviated Absolute Handgrip Strength Percentiles by Gender and Ethnicity.

Variables	Percentile (kilograms)								
	10	20	30	40	50	60	70	80	90
**Males**									

*Non-Hispanic Asian*									

10 Years Old	14.1	16.2	17.2	18.2	19.4	21.0	22.0	22.8	25.2
20 Years Old	32.6	35.4	37.4	39.6	41.7	43.3	44.9	47.2	51.0
30 Years Old	36.2	38.5	41.4	43.1	45.5	47.0	49.4	51.6	54.2
40 Years Old	36.4	39.2	41.5	43.1	44.7	46.1	48.5	50.8	53.4
50 Years Old	35.5	38.3	40.0	41.8	42.9	44.5	46.5	48.7	51.3
60 Years Old	32.8	35.0	36.8	38.7	40.2	42.1	43.5	45.5	47.4
70 Years Old	28.1	29.7	32.1	33.6	35.4	37.4	38.7	40.6	42.8

*Non-Hispanic Black*									

10 Years Old	15.9	18.1	19.9	21.1	22.3	23.6	24.6	26.2	27.9
20 Years Old	36.8	40.8	43.7	46.7	49.6	51.6	53.4	56.5	59.6
30 Years Old	42.3	46.4	49.4	51.9	54.2	56.8	59.1	62.3	66.0
40 Years Old	42.8	45.6	48.3	50.2	52.3	54.6	57.0	59.4	63.5
50 Years Old	40.3	42.9	45.4	47.1	49.4	51.3	53.7	55.9	60.0
60 Years Old	35.1	39.3	41.9	43.6	46.0	48.0	50.7	53.2	57.0
70 Years Old	27.9	34.4	37.2	39.1	41.2	43.8	46.4	49.2	52.7

*Hispanic*									

10 Years Old	15.0	16.6	17.7	18.8	19.9	20.9	22.2	23.7	25.9
20 Years Old	34.5	38.4	40.9	42.9	45.6	48.2	50.7	53.8	56.9
30 Years Old	39.5	43.2	45.7	47.0	49.5	51.9	53.8	57.0	60.6
40 Years Old	38.7	41.6	43.8	45.2	46.9	48.8	50.6	53.4	57.2
50 Years Old	35.5	38.2	40.4	42.4	43.7	45.5	47.7	50.1	53.8
60 Years Old	31.1	34.5	36.7	39.1	40.8	42.9	45.3	47.5	51.0
70 Years Old	26.9	30.2	32.3	34.5	36.9	39.0	41.3	43.3	46.5

*Non-Hispanic White*									

10 Years Old	14.6	16.6	17.8	19.0	20.1	21.5	22.6	25.0	26.8
20 Years Old	37.1	40.2	42.5	44.8	46.6	48.9	51.2	55.0	59.4
30 Years Old	42.8	46.2	48.4	50.5	52.5	54.4	56.8	59.9	63.7
40 Years Old	41.1	44.9	47.4	49.4	51.0	52.8	54.8	57.2	60.5
50 Years Old	38.0	42.1	44.8	46.9	48.3	50.0	51.8	53.9	57.2
60 Years Old	35.2	39.3	41.9	44.0	45.4	47.1	49.1	50.9	54.3
70 Years Old	32.0	35.3	37.6	39.7	41.4	43.1	45.2	46.9	50.1

**Females**									

*Non-Hispanic Asian*									

10 Years Old	13.6	15.0	15.8	16.5	17.5	18.4	19.2	20.3	22.5
20 Years Old	22.1	23.9	25.1	26.0	27.0	28.6	29.8	31.5	33.5
30 Years Old	22.4	24.0	25.1	25.8	27.2	28.1	29.3	32.0	34.0
40 Years Old	22.7	24.3	25.7	27.0	28.3	29.2	30.4	32.8	34.6
50 Years Old	22.9	24.4	25.9	27.4	28.6	29.9	30.9	32.5	34.3
60 Years Old	21.5	22.8	24.2	25.3	26.4	27.9	28.7	29.8	31.9
70 Years Old	17.6	19.5	20.6	21.3	22.9	24.5	25.2	26.1	28.5

*Non-Hispanic Black*									

10 Years Old	16.3	17.9	19.2	19.9	21.1	22.3	23.4	25.1	27.2
20 Years Old	26.7	28.9	30.4	31.7	33.3	34.7	36.8	38.8	42.2
30 Years Old	28.0	30.3	31.8	33.6	35.1	36.6	38.6	40.8	43.3
40 Years Old	26.8	29.8	31.2	33.0	34.6	35.9	37.8	39.9	42.6
50 Years Old	24.9	28.5	30.0	31.6	33.2	34.4	36.1	37.9	41.3
60 Years Old	22.8	25.9	27.9	29.4	30.7	32.2	33.4	34.9	38.4
70 Years Old	20.1	22.1	24.5	26.1	27.0	28.8	29.9	31.4	34.1

*Hispanic*									

10 Years Old	14.2	15.5	16.7	17.4	18.1	18.9	20.3	21.6	23.8
20 Years Old	23.8	25.9	27.3	28.9	29.9	31.0	32.3	34.0	36.0
30 Years Old	24.7	26.8	28.0	29.5	30.5	31.5	32.6	34.4	36.3
40 Years Old	23.9	26.2	27.5	28.6	29.5	30.7	32.0	33.7	36.3
50 Years Old	22.6	24.9	26.2	27.4	28.3	29.6	31.1	32.7	35.2
60 Years Old	20.4	22.4	23.7	25.2	26.4	27.4	28.8	30.4	32.1
70 Years Old	17.5	19.0	20.4	22.1	23.3	23.9	25.1	26.7	27.8

*Non-Hispanic White*									

10 Years Old	14.5	15.7	16.9	18.1	18.8	19.5	20.5	22.0	23.4
20 Years Old	25.3	27.5	29.0	29.8	31.1	32.2	33.3	35.0	37.7
30 Years Old	26.9	28.8	30.1	31.3	32.2	33.3	34.5	36.3	38.9
40 Years Old	26.2	28.2	29.4	30.8	31.7	23.7	24.0	36.0	38.9
50 Years Old	25.0	27.2	28.4	29.7	30.7	31.7	33.0	34.9	37.6
60 Years Old	23.2	25.3	26.5	27.4	28.7	29.6	30.9	32.5	34.1
70 Years Old	20.0	22.0	23.1	23.8	25.1	26.1	27.4	28.7	29.8

**Table 4.** Abbreviated Body Mass Index Normalized Handgrip Strength Percentiles by Gender and Ethnicity.

Variables	Percentile								
	10	20	30	40	50	60	70	80	90
**Males**									

*Non-Hispanic Asian*									

10 Years Old	0.79	0.85	0.90	0.94	1.00	1.03	1.14	1.25	1.37
20 Years Old	1.30	1.53	1.61	1.72	1.79	1.89	1.99	2.12	2.31
30 Years Old	1.35	1.54	1.66	1.77	1.85	1.95	2.03	2.14	2.30
40 Years Old	1.39	1.49	1.63	1.72	1.81	1.88	1.96	2.03	2.19
50 Years Old	1.39	1.45	1.58	1.66	1.74	1.81	1.88	1.96	2.10
60 Years Old	1.28	1.37	1.46	1.55	1.63	1.70	1.76	1.88	1.98
70 Years Old	1.06	1.19	1.27	1.36	1.45	1.52	1.58	1.71	1.77

*Non-Hispanic Black*									

10 Years Old	0.86	0.94	1.01	1.06	1.13	1.21	1.28	1.34	1.44
20 Years Old	1.28	1.51	1.66	1.81	1.94	2.06	2.20	2.33	2.53
30 Years Old	1.35	1.55	1.66	1.81	1.92	2.05	2.20	2.34	2.54
40 Years Old	1.31	1.47	1.59	1.72	1.82	1.92	2.05	2.19	2.37
50 Years Old	1.21	1.37	1.50	1.62	1.73	1.81	1.93	2.08	2.25
60 Years Old	1.06	1.25	1.38	1.50	1.62	1.70	1.82	2.00	2.17
70 Years Old	0.89	1.13	1.24	1.36	1.47	1.56	1.69	1.86	2.02

*Hispanic*									

10 Years Old	0.70	0.79	0.85	0.93	0.99	1.05	1.12	1.19	1.30
20 Years Old	1.22	1.40	1.54	1.65	1.79	1.90	2.01	2.13	2.31
30 Years Old	1.22	1.40	1.52	1.60	1.72	1.83	1.95	2.08	2.22
40 Years Old	1.22	1.35	1.45	1.52	1.60	1.70	1.78	1.93	2.09
50 Years Old	1.18	1.30	1.38	1.46	1.52	1.61	1.68	1.82	2.00
60 Years Old	1.06	1.19	1.27	1.34	1.42	1.51	1.59	1.71	1.86
70 Years Old	0.90	1.05	1.13	1.18	1.27	1.35	1.46	1.53	1.62

*Non-Hispanic White*									

10 Years Old	0.78	0.89	0.95	1.02	1.07	1.15	1.23	1.32	1.44
20 Years Old	1.36	1.55	1.67	1.81	1.94	2.06	2.16	2.28	2.50
30 Years Old	1.38	1.54	1.70	1.82	1.93	2.04	2.12	2.23	2.46
40 Years Old	1.30	1.43	1.59	1.71	1.80	1.89	1.96	2.06	2.26
50 Years Old	1.21	1.36	1.48	1.61	1.70	1.78	1.86	1.95	2.13
60 Years Old	1.13	1.28	1.40	1.51	1.60	1.67	1.77	1.89	2.04
70 Years Old	1.03	1.18	1.29	1.37	1.46	1.53	1.63	1.76	1.89

**Females**									

*Non-Hispanic Asian*									

10 Years Old	0.73	0.79	0.86	0.89	0.94	0.98	1.04	1.14	1.22
20 Years Old	0.96	1.09	1.16	1.23	1.29	1.33	1.41	1.48	1.62
30 Years Old	0.91	1.01	1.08	1.14	1.21	1.28	1.36	1.43	1.55
40 Years Old	0.88	0.97	1.04	1.11	1.18	1.27	1.34	1.42	1.53
50 Years Old	0.86	0.95	1.02	1.10	1.16	1.24	1.31	1.38	1.48
60 Years Old	0.79	0.89	0.94	1.01	1.08	1.13	1.21	1.25	1.34
70 Years Old	0.64	0.76	0.82	0.86	0.95	0.98	1.06	1.11	1.19

*Non-Hispanic Black*									

10 Years Old	0.72	0.84	0.92	1.00	1.07	1.11	1.17	1.23	1.34
20 Years Old	0.90	1.02	1.11	1.19	1.25	1.31	1.40	1.49	1.64
30 Years Old	0.77	0.90	0.98	1.04	1.10	1.18	1.23	1.36	1.52
40 Years Old	0.68	0.80	0.90	0.97	1.05	1.13	1.20	1.32	1.47
50 Years Old	0.65	0.77	0.86	0.94	1.03	1.12	1.19	1.29	1.44
60 Years Old	0.64	0.75	0.82	0.89	0.97	1.04	1.10	1.18	1.36
70 Years Old	0.59	0.70	0.76	0.80	0.86	0.91	0.96	1.04	1.23

*Hispanic*									

10 Years Old	0.66	0.74	0.80	0.86	0.90	0.94	1.01	1.06	1.16
20 Years Old	0.83	0.94	1.03	1.09	1.16	1.23	1.31	1.42	1.53
30 Years Old	0.74	0.86	0.93	0.99	1.05	1.12	1.20	1.31	1.41
40 Years Old	0.71	0.82	0.87	0.94	0.99	1.06	1.15	1.23	1.37
50 Years Old	0.69	0.77	0.82	0.89	0.93	1.00	1.10	1.17	1.32
60 Years Old	0.62	0.68	0.74	0.79	0.85	0.91	0.99	1.06	1.18
70 Years Old	0.54	0.58	0.64	0.69	0.76	0.80	0.84	0.94	0.99

*Non-Hispanic White*									

10 Years Old	0.76	0.84	0.89	0.94	0.98	1.02	1.07	1.14	1.23
20 Years Old	0.84	1.02	1.11	1.21	1.28	1.36	1.43	1.48	1.59
30 Years Old	0.81	0.96	1.06	1.15	1.22	1.29	1.36	1.44	1.57
40 Years Old	0.78	0.90	1.01	1.09	1.17	1.25	1.32	1.40	1.54
50 Years Old	0.74	0.85	0.95	1.03	1.12	1.19	1.26	1.36	1.49
60 Years Old	0.69	0.78	0.86	0.94	1.02	1.08	1.13	1.24	1.37
70 Years Old	0.61	0.69	0.75	0.82	0.88	0.92	0.97	1.08	1.20

**Table 5.** Abbreviated Dominant Absolute Handgrip Strength Percentiles by Gender and Ethnicity.

Variables	Percentile (kilograms)								
	10	20	30	40	50	60	70	80	90
**Males**									

*Non-Hispanic Asian*									

10 Years Old	14.1	15.6	16.8	17.9	19.0	20.2	21.6	22.6	24.4
20 Years Old	32.2	35.1	37.0	38.5	41.1	42.8	44.5	47.1	51.1
30 Years Old	36.3	38.4	40.7	42.7	45.0	46.7	49.1	51.5	55.2
40 Years Old	36.1	38.0	41.2	43.0	44.6	45.9	48.3	50.2	53.5
50 Years Old	34.6	36.5	40.0	41.5	43.0	44.1	46.1	47.8	50.7
60 Years Old	31.9	33.5	36.2	37.9	39.8	41.1	42.8	44.5	47.1
70 Years Old	27.7	29.0	30.8	32.5	34.6	36.0	37.5	39.5	42.3

*Non-Hispanic Black*									

10 Years Old	15.5	17.7	19.3	20.9	21.8	22.8	23.9	26.0	27.5
20 Years Old	36.1	40.5	43.1	45.8	48.3	50.4	52.9	55.5	58.9
30 Years Old	41.2	45.6	48.2	50.9	53.0	55.6	58.3	60.7	65.3
40 Years Old	40.7	44.6	47.4	49.5	51.0	53.8	56.0	58.5	63.0
50 Years Old	37.7	41.6	44.6	46.4	48.0	50.6	52.7	55.6	59.4
60 Years Old	33.1	37.7	40.5	42.5	44.6	46.9	49.7	52.7	55.9
70 Years Old	27.0	32.4	35.3	37.6	40.2	42.4	45.4	48.2	50.7

*Hispanic*									

10 Years Old	14.7	16.2	17.3	18.4	19.5	20.6	21.8	23.1	25.3
20 Years Old	33.6	37.4	40.3	42.4	44.9	47.7	49.5	53.2	56.6
30 Years Old	38.0	42.0	44.9	46.6	48.4	51.1	52.9	56.3	60.3
40 Years Old	37.2	40.7	43.0	44.6	46.1	47.7	50.0	52.4	56.5
50 Years Old	34.2	37.8	39.9	41.6	43.2	44.4	47.1	49.2	52.9
60 Years Old	30.0	34.0	36.7	38.4	40.2	42.1	44.4	47.2	50.2
70 Years Old	26.0	29.6	32.2	34.1	35.8	38.6	39.8	43.3	46.1

*Non-Hispanic White*									

10 Years Old	14.5	16.1	17.6	18.7	19.7	21.3	22.4	24.6	26.5
20 Years Old	36.3	39.6	42.0	44.1	46.2	48.5	50.7	54.1	58.9
30 Years Old	41.5	45.2	47.6	49.2	51.7	53.6	56.1	59.1	63.0
40 Years Old	39.6	43.7	46.2	48.1	50.1	51.8	54.0	56.5	60.0
50 Years Old	36.7	40.9	43.5	45.8	47.4	48.9	51.0	53.2	56.8
60 Years Old	34.3	37.9	40.7	43.0	44.8	46.1	48.2	50.4	53.8
70 Years Old	31.2	33.9	36.7	38.7	40.9	42.3	44.5	46.6	49.4

**Females**									

*Non-Hispanic Asian*									

10 Years Old	13.5	14.7	15.5	16.3	17.0	18.1	19.0	20.6	22.4
20 Years Old	21.7	23.8	24.9	25.4	26.8	28.3	29.6	31.1	32.8
30 Years Old	21.6	23.6	24.9	25.4	26.8	28.0	29.0	31.5	33.3
40 Years Old	21.9	23.9	25.2	26.3	27.7	28.9	30.1	32.3	34.3
50 Years Old	22.3	24.2	25.4	26.7	27.9	29.3	30.7	32.0	34.4
60 Years Old	21.2	22.8	23.8	24.8	25.6	27.3	28.5	29.4	31.8
70 Years Old	17.0	19.2	20.1	21.1	22.2	24.2	25.1	25.9	28.1

*Non-Hispanic Black*									

10 Years Old	15.8	17.5	18.9	19.8	20.9	22.1	23.0	25.0	27.2
20 Years Old	26.3	28.7	30.0	31.2	32.9	34.4	36.3	38.5	41.7
30 Years Old	27.1	29.7	31.1	32.8	34.8	36.1	37.8	40.3	42.8
40 Years Old	25.9	28.6	30.5	32.5	34.3	35.6	37.0	39.6	42.1
50 Years Old	24.0	26.9	29.3	31.3	32.9	34.2	35.4	37.6	40.7
60 Years Old	21.5	24.7	27.1	28.8	30.4	31.7	32.9	34.7	38.0
70 Years Old	18.3	21.4	23.5	25.0	26.5	27.7	29.3	31.0	33.8

*Hispanic*									

10 Years Old	14.0	15.1	16.3	17.1	17.9	18.6	20.0	21.6	23.7
20 Years Old	23.5	25.6	27.1	28.6	29.6	30.8	32.2	33.7	35.9
30 Years Old	24.2	26.2	27.9	29.2	30.3	31.3	32.3	34.3	36.2
40 Years Old	23.3	25.4	27.2	28.1	29.1	30.3	31.6	33.6	36.1
50 Years Old	21.7	23.9	25.8	26.7	27.8	29.1	30.7	32.5	35.1
60 Years Old	19.6	21.6	23.2	24.6	25.8	27.1	28.6	30.3	32.1
70 Years Old	17.1	18.5	19.9	21.6	22.9	23.7	24.9	26.7	27.9

*Non-Hispanic White*									

10 Years Old	14.3	15.5	16.8	17.8	18.7	19.4	20.4	21.6	23.4
20 Years Old	24.9	27.2	28.7	29.7	30.9	31.9	33.2	34.6	37.6
30 Years Old	26.3	28.3	29.7	31.0	32.0	33.2	34.4	35.9	38.6
40 Years Old	25.3	27.6	29.1	30.4	31.4	32.6	33.7	35.6	38.5
50 Years Old	23.9	26.5	28.1	29.2	30.4	31.4	32.6	34.6	37.3
60 Years Old	22.1	24.6	26.0	27.1	28.2	29.3	30.5	32.1	34.1
70 Years Old	19.1	21.3	22.5	23.6	24.6	25.7	27.1	28.3	29.6

**Table 6.** Abbreviated Non-Dominant Absolute Handgrip Strength Percentiles by Gender and Ethnicity.

Variables	Percentile (kilograms)								
	10	20	30	40	50	60	70	80	90
**Males**									

*Non-Hispanic Asian*									

10 Years Old	12.6	13.8	15.8	17.0	17.9	19.0	20.2	22.0	23.7
20 Years Old	29.7	32.4	34.3	36.6	38.5	40.3	41.9	44.5	46.9
30 Years Old	32.5	36.2	37.6	39.7	41.5	43.2	45.1	48.4	51.3
40 Years Old	33.8	36.4	38.2	39.8	41.2	42.7	44.7	47.0	50.8
50 Years Old	33.3	35.2	37.1	38.6	39.8	41.4	43.3	44.7	48.0
60 Years Old	29.7	32.1	33.7	35.3	36.9	38.6	40.1	42.1	43.7
70 Years Old	24.3	27.2	28.7	30.3	32.3	33.8	35.3	38.3	39.7

*Non-Hispanic Black*									

10 Years Old	15.3	16.7	18.3	19.5	20.8	21.9	23.4	24.4	26.6
20 Years Old	33.6	37.0	40.4	43.2	45.4	47.7	49.9	52.6	56.3
30 Years Old	39.0	41.9	45.8	49.4	51.3	53.2	55.8	58.7	62.6
40 Years Old	38.7	41.8	44.7	48.0	49.5	51.5	53.8	56.5	60.6
50 Years Old	35.3	39.2	41.3	44.1	45.8	48.4	50.3	53.0	57.1
60 Years Old	30.0	34.9	37.1	39.9	42.1	45.0	47.1	49.9	53.5
70 Years Old	24.5	29.7	32.6	35.4	38.1	40.3	43.0	45.7	48.7

*Hispanic*									

10 Years Old	13.6	15.1	16.5	17.5	18.6	19.7	20.8	22.0	24.6
20 Years Old	32.5	35.8	37.9	40.2	42.7	44.8	47.1	50.2	53.9
30 Years Old	37.1	40.1	42.5	44.4	46.1	48.1	50.6	53.5	57.2
40 Years Old	35.7	38.6	40.9	42.4	43.8	45.7	47.7	50.4	54.2
50 Years Old	32.3	35.4	37.7	39.3	41.3	42.9	44.7	47.4	50.8
60 Years Old	28.4	31.7	33.9	36.2	38.7	39.9	42.2	44.9	47.5
70 Years Old	23.9	27.6	29.5	32.0	34.4	35.2	38.6	40.7	43.0

*Non-Hispanic White*									

10 Years Old	13.7	15.2	16.2	17.7	19.0	20.4	21.4	23.4	26.2
20 Years Old	33.4	36.9	39.5	41.6	43.8	45.8	48.2	51.2	55.0
30 Years Old	39.2	42.4	45.1	47.0	49.0	51.3	53.3	55.5	59.5
40 Years Old	38.2	42.1	44.7	46.5	48.0	49.9	51.9	54.0	57.7
50 Years Old	35.5	39.8	42.6	44.4	45.8	47.4	49.3	51.6	55.0
60 Years Old	32.8	36.7	39.3	41.3	43.1	44.6	46.1	48.5	51.7
70 Years Old	29.4	32.3	34.6	36.8	38.9	40.6	41.9	44.0	46.9

**Females**									

*Non-Hispanic Asian*									

10 Years Old	12.6	13.5	14.4	15.0	16.0	16.8	17.9	19.0	20.0
20 Years Old	19.7	21.2	22.9	23.9	24.9	26.1	27.8	29.1	31.5
30 Years Old	20.3	21.7	23.0	23.7	25.0	26.2	28.1	29.3	31.8
40 Years Old	21.3	22.5	23.8	24.8	25.7	27.1	28.9	30.2	32.2
50 Years Old	21.0	22.7	24.2	25.4	26.1	27.5	28.9	30.2	31.8
60 Years Old	18.0	21.0	22.5	23.6	24.6	25.7	26.6	27.7	29.4
70 Years Old	13.8	17.5	18.7	19.8	21.6	22.4	23.0	23.7	26.2

*Non-Hispanic Black*									

10 Years Old	14.8	16.1	17.5	18.7	19.6	20.4	21.9	23.3	25.2
20 Years Old	24.0	26.2	27.9	29.4	30.6	32.0	33.9	36.0	38.5
30 Years Old	24.8	27.8	29.1	30.6	32.0	33.9	35.4	37.3	39.7
40 Years Old	24.0	26.9	28.5	30.0	31.6	33.3	34.8	36.7	39.1
50 Years Old	22.9	25.4	27.3	28.9	30.4	31.7	33.3	35.3	37.7
60 Years Old	21.1	23.5	25.4	27.0	28.2	29.4	30.9	32.7	34.9
70 Years Old	18.3	20.7	22.3	23.9	25.2	26.5	28.0	29.2	30.9

*Hispanic*									

10 Years Old	12.9	14.2	15.3	16.1	16.8	17.8	18.9	20.2	21.9
20 Years Old	21.8	23.2	24.7	26.2	27.5	28.8	29.7	31.2	33.6
30 Years Old	22.7	24.3	25.8	27.1	28.1	29.2	30.3	31.9	34.3
40 Years Old	21.4	23.7	25.5	26.7	27.5	28.6	30.0	31.7	34.1
50 Years Old	19.9	22.5	24.4	25.6	26.5	27.6	29.0	30.6	33.0
60 Years Old	18.3	20.5	22.1	23.3	24.5	25.5	26.6	27.9	30.2
70 Years Old	16.1	17.5	18.7	19.7	21.1	22.1	23.3	24.1	25.7

*Non-Hispanic White*									

10 Years Old	12.8	14.5	15.7	16.4	17.4	18.1	18.7	20.1	22.2
20 Years Old	22.8	24.7	26.2	27.3	28.6	29.5	31.0	32.3	35.0
30 Years Old	24.3	26.1	27.6	28.8	29.8	30.9	32.0	33.4	36.4
40 Years Old	23.6	25.7	27.1	28.3	29.4	30.6	31.7	33.2	35.9
50 Years Old	22.4	24.8	26.2	27.2	28.4	29.7	31.0	32.5	34.6
60 Years Old	20.9	23.2	24.5	25.4	26.3	27.5	28.9	30.3	32.0
70 Years Old	18.0	20.1	21.4	22.3	23.0	24.1	25.3	26.7	28.4

**Table 7.** Abbreviated Body Mass Index Normalized Dominant Handgrip Strength Percentiles by Gender and Ethnicity.

Variables	Percentile								
	10	20	30	40	50	60	70	80	90
**Males**									

*Non-Hispanic Asian*									

10 Years Old	0.79	0.84	0.89	0.94	0.98	1.03	1.13	1.22	1.36
20 Years Old	1.27	1.50	1.58	1.70	1.76	1.87	1.97	2.10	2.28
30 Years Old	1.32	1.51	1.65	1.75	1.83	1.93	2.02	2.12	2.29
40 Years Old	1.36	1.47	1.62	1.70	1.78	1.85	1.95	2.02	2.19
50 Years Old	1.35	1.44	1.55	1.63	1.71	1.77	1.87	1.94	2.10
60 Years Old	1.23	1.35	1.42	1.51	1.59	1.68	1.74	1.86	1.97
70 Years Old	1.00	1.15	1.24	1.33	1.42	1.51	1.56	1.69	1.75

*Non-Hispanic Black*									

10 Years Old	0.83	0.91	0.98	1.04	1.10	1.17	1.24	1.33	1.42
20 Years Old	1.26	1.48	1.64	1.78	1.87	2.02	2.17	2.30	2.49
30 Years Old	1.34	1.53	1.63	1.76	1.88	2.01	2.15	2.31	2.51
40 Years Old	1.29	1.43	1.53	1.68	1.79	1.88	1.99	2.18	2.33
50 Years Old	1.18	1.32	1.44	1.59	1.69	1.77	1.87	2.07	2.20
60 Years Old	1.03	1.22	1.34	1.46	1.55	1.65	1.77	1.95	2.11
70 Years Old	0.88	1.10	1.21	1.30	1.39	1.52	1.65	1.79	1.97

*Hispanic*									

10 Years Old	0.68	0.77	0.84	0.91	0.98	1.04	1.09	1.16	1.28
20 Years Old	1.18	1.36	1.53	1.64	1.75	1.85	1.97	2.11	2.28
30 Years Old	1.16	1.36	1.50	1.58	1.67	1.78	1.92	2.05	2.21
40 Years Old	1.18	1.33	1.43	1.50	1.56	1.67	1.76	1.89	2.08
50 Years Old	1.15	1.29	1.36	1.43	1.50	1.59	1.66	1.79	1.98
60 Years Old	1.02	1.16	1.25	1.32	1.41	1.48	1.57	1.69	1.84
70 Years Old	0.85	1.00	1.10	1.15	1.24	1.31	1.42	1.52	1.61

*Non-Hispanic White*									

10 Years Old	0.77	0.85	0.93	1.00	1.06	1.13	1.20	1.30	1.43
20 Years Old	1.35	1.52	1.65	1.80	1.93	2.04	2.15	2.27	2.48
30 Years Old	1.38	1.53	1.66	1.79	1.91	2.00	2.10	2.22	2.45
40 Years Old	1.26	1.42	1.55	1.66	1.75	1.85	1.92	2.03	2.24
50 Years Old	1.15	1.33	1.45	1.55	1.65	1.74	1.82	1.93	2.10
60 Years Old	1.07	1.24	1.35	1.45	1.57	1.64	1.73	1.86	2.01
70 Years Old	0.98	1.14	1.24	1.33	1.45	1.50	1.60	1.74	1.87

**Females**									

*Non-Hispanic Asian*									

10 Years Old	0.71	0.77	0.84	0.88	0.92	0.99	1.04	1.11	1.23
20 Years Old	0.96	1.09	1.16	1.22	1.27	1.31	1.39	1.47	1.60
30 Years Old	0.89	1.00	1.07	1.13	1.19	1.27	1.33	1.43	1.52
40 Years Old	0.85	0.94	1.03	1.09	1.17	1.25	1.32	1.42	1.51
50 Years Old	0.83	0.92	1.01	1.07	1.15	1.22	1.29	1.37	1.47
60 Years Old	0.76	0.88	0.94	0.99	1.05	1.12	1.18	1.25	1.33
70 Years Old	0.61	0.75	0.79	0.85	0.91	0.98	1.05	1.11	1.18

*Non-Hispanic Black*									

10 Years Old	0.71	0.82	0.89	0.99	1.04	1.10	1.15	1.23	1.35
20 Years Old	0.88	0.99	1.09	1.18	1.25	1.31	1.37	1.47	1.61
30 Years Old	0.75	0.86	0.97	1.03	1.08	1.17	1.22	1.33	1.49
40 Years Old	0.66	0.79	0.89	0.96	1.03	1.12	1.18	1.29	1.46
50 Years Old	0.63	0.76	0.84	0.93	1.02	1.10	1.17	1.26	1.43
60 Years Old	0.61	0.72	0.79	0.87	0.96	1.02	1.09	1.17	1.33
70 Years Old	0.57	0.65	0.72	0.78	0.84	0.89	0.95	1.03	1.20

*Hispanic*									

10 Years Old	0.65	0.72	0.79	0.85	0.90	0.94	0.99	1.05	1.16
20 Years Old	0.82	0.94	1.01	1.08	1.15	1.23	1.30	1.40	1.52
30 Years Old	0.74	0.85	0.90	0.97	1.03	1.11	1.19	1.30	1.41
40 Years Old	0.70	0.80	0.85	0.92	0.97	1.03	1.13	1.23	1.37
50 Years Old	0.66	0.75	0.81	0.87	0.93	0.98	1.08	1.16	1.32
60 Years Old	0.60	0.68	0.73	0.77	0.84	0.89	0.97	1.05	1.17
70 Years Old	0.51	0.57	0.63	0.67	0.74	0.80	0.84	0.91	0.98

*Non-Hispanic White*									

10 Years Old	0.73	0.81	0.87	0.93	0.98	1.02	1.06	1.13	1.22
20 Years Old	0.84	0.99	1.10	1.19	1.26	1.35	1.42	1.48	1.59
30 Years Old	0.79	0.95	1.04	1.13	1.20	1.28	1.35	1.42	1.57
40 Years Old	0.75	0.89	0.99	1.07	1.16	1.23	1.30	1.40	1.53
50 Years Old	0.72	0.84	0.94	1.01	1.10	1.17	1.25	1.36	1.47
60 Years Old	0.66	0.76	0.85	0.92	1.00	1.06	1.13	1.24	1.36
70 Years Old	0.59	0.68	0.74	0.81	0.86	0.91	0.96	1.07	1.19

**Table 8.** Abbreviated Body Mass Index Normalized Non-Dominant Handgrip Strength Percentiles by Gender and Ethnicity.

Variables	Percentile								
	10	20	30	40	50	60	70	80	90
**Males**									

*Non-Hispanic Asian*									

10 Years Old	0.71	0.78	0.81	0.86	0.91	0.99	1.07	1.13	1.33
20 Years Old	1.18	1.37	1.49	1.59	1.65	1.73	1.85	1.95	2.12
30 Years Old	1.20	1.37	1.53	1.64	1.70	1.78	1.88	1.96	2.11
40 Years Old	1.26	1.38	1.52	1.60	1.65	1.74	1.82	1.89	2.04
50 Years Old	1.28	1.38	1.47	1.53	1.60	1.68	1.75	1.82	1.96
60 Years Old	1.17	1.26	1.34	1.41	1.50	1.58	1.64	1.71	1.82
70 Years Old	0.96	1.03	1.15	1.24	1.35	1.41	1.46	1.53	1.64

*Non-Hispanic Black*									

10 Years Old	0.77	0.86	0.93	0.98	1.05	1.14	1.19	1.29	1.34
20 Years Old	1.17	1.39	1.53	1.66	1.79	1.90	2.04	2.19	2.36
30 Years Old	1.25	1.40	1.56	1.69	1.80	1.89	2.06	2.22	2.38
40 Years Old	1.22	1.35	1.46	1.58	1.67	1.78	1.94	2.10	2.20
50 Years Old	1.11	1.26	1.36	1.46	1.55	1.68	1.82	1.98	2.09
60 Years Old	0.96	1.13	1.26	1.36	1.46	1.57	1.69	1.85	2.03
70 Years Old	0.80	0.99	1.16	1.26	1.36	1.43	1.54	1.68	1.92

*Hispanic*									

10 Years Old	0.63	0.74	0.80	0.86	0.92	0.99	1.05	1.12	1.23
20 Years Old	1.14	1.28	1.42	1.53	1.65	1.76	1.88	2.01	2.18
30 Years Old	1.14	1.28	1.39	1.50	1.58	1.70	1.82	1.94	2.13
40 Years Old	1.14	1.26	1.34	1.42	1.49	1.59	1.69	1.79	2.00
50 Years Old	1.10	1.20	1.29	1.36	1.44	1.51	1.60	1.70	1.90
60 Years Old	0.96	1.06	1.17	1.25	1.34	1.40	1.50	1.59	1.77
70 Years Old	0.78	0.91	1.02	1.10	1.17	1.24	1.34	1.43	1.56

*Non-Hispanic White*									

10 Years Old	0.73	0.81	0.88	0.92	0.99	1.07	1.17	1.25	1.37
20 Years Old	1.21	1.43	1.56	1.69	1.80	1.93	2.03	2.14	2.34
30 Years Old	1.26	1.45	1.57	1.70	1.80	1.91	1.99	2.10	2.28
40 Years Old	1.20	1.36	1.48	1.61	1.70	1.78	1.87	1.96	2.13
50 Years Old	1.12	1.28	1.39	1.53	1.62	1.69	1.78	1.87	2.03
60 Years Old	1.02	1.19	1.30	1.43	1.51	1.58	1.69	1.80	1.94
70 Years Old	0.92	1.08	1.18	1.28	1.35	1.43	1.53	1.67	1.77

**Females**									

*Non-Hispanic Asian*									

10 Years Old	0.68	0.73	0.77	0.82	0.85	0.92	0.97	1.00	1.16
20 Years Old	0.85	0.94	1.06	1.11	1.18	1.24	1.31	1.40	1.51
30 Years Old	0.83	0.89	1.00	1.06	1.12	1.18	1.27	1.36	1.47
40 Years Old	0.81	0.90	0.97	1.04	1.10	1.15	1.24	1.31	1.42
50 Years Old	0.76	0.88	0.95	1.02	1.08	1.13	1.21	1.27	1.36
60 Years Old	0.65	0.79	0.87	0.95	0.99	1.06	1.12	1.19	1.25
70 Years Old	0.52	0.64	0.74	0.82	0.85	0.94	1.01	1.07	1.10

*Non-Hispanic Black*									

10 Years Old	0.66	0.78	0.86	0.92	0.99	1.04	1.08	1.14	1.25
20 Years Old	0.81	0.94	1.00	1.08	1.15	1.20	1.29	1.38	1.52
30 Years Old	0.70	0.82	0.90	0.96	1.01	1.06	1.15	1.25	1.40
40 Years Old	0.63	0.75	0.83	0.88	0.96	1.02	1.10	1.22	1.39
50 Years Old	0.60	0.71	0.79	0.85	0.94	1.02	1.09	1.20	1.36
60 Years Old	0.58	0.67	0.74	0.82	0.90	0.96	1.02	1.10	1.23
70 Years Old	0.52	0.62	0.70	0.76	0.81	0.84	0.91	0.97	1.08

*Hispanic*									

10 Years Old	0.60	0.69	0.74	0.79	0.84	0.88	0.92	0.99	1.11
20 Years Old	0.76	0.85	0.92	0.98	1.07	1.14	1.20	1.29	1.42
30 Years Old	0.70	0.78	0.85	0.92	0.99	1.05	1.12	1.19	1.30
40 Years Old	0.66	0.74	0.81	0.88	0.94	0.99	1.07	1.14	1.25
50 Years Old	0.62	0.70	0.76	0.82	0.88	0.93	1.02	1.10	1.21
60 Years Old	0.56	0.64	0.68	0.74	0.79	0.85	0.93	0.99	1.10
70 Years Old	0.49	0.55	0.58	0.64	0.69	0.75	0.79	0.84	0.92

*Non-Hispanic White*									

10 Years Old	0.68	0.77	0.81	0.86	0.90	0.95	1.01	1.06	1.14
20 Years Old	0.79	0.93	1.02	1.10	1.17	1.23	1.30	1.38	1.46
30 Years Old	0.75	0.87	0.97	1.05	1.12	1.19	1.26	1.34	1.43
40 Years Old	0.71	0.82	0.92	1.01	1.08	1.15	1.23	1.32	1.42
50 Years Old	0.67	0.77	0.87	0.95	1.03	1.10	1.19	1.27	1.39
60 Years Old	0.61	0.71	0.79	0.86	0.93	1.00	1.08	1.17	1.27
70 Years Old	0.54	0.63	0.69	0.75	0.81	0.85	0.92	1.01	1.11

## ABBREVIATIONS

BMI: Body Mass Index
HGS: Handgrip Strength
NHANES: National Health and Nutrition Examination Survey
